# A High-Efficiency Si Nanowire Array/Perovskite Hybrid Solar Cell

**DOI:** 10.1186/s11671-016-1785-y

**Published:** 2017-01-05

**Authors:** Xin Yan, Chen Zhang, Jiamin Wang, Xia Zhang, Xiaomin Ren

**Affiliations:** State Key Laboratory of Information Photonics and Optical Communications, Beijing University of Posts and Telecommunications, Beijing, 100876 China

**Keywords:** Nanowire, Perovskite, Solar cell, Si, 62.23.Hj, 84.60.Jt, 61.72.uf

## Abstract

A low-cost Si nanowire array/perovskite hybrid solar cell is proposed and simulated. The solar cell consists of a Si p-i-n nanowire array filled with CH_3_NH_3_PbI_3_, in which both the nanowires and perovskite absorb the incident light while the nanowires act as the channels for transporting photo-generated electrons and holes. The hybrid structure has a high absorption efficiency in a broad wavelength range of 300~800 nm. A large short-circuit current density of 28.8 mA/cm^2^ and remarkable conversion efficiency of 13.3% are obtained at a thin absorber thickness of 1.6 μm, which are comparable to the best results of III–V nanowire solar cells.

## Background

Semiconductor nanowires (NWs) have attracted great attention as building blocks for future electronic and photonic devices [1]. Due to the advantages like light-trapping and light-concentrating, which can dramatically enhance the absorption of light, the ability of strain relaxation, new charge separation mechanisms, and low filling ratio, NWs have shown great potential in low-cost high-performance solar cells [2–6]. Silicon (Si) NW solar cells are particularly promising due to the low cost, abundance in nature, nontoxicity, and long-term stability and have been widely reported in recent years [7–10]. However, due to its indirect bandgap, Si has a poor absorption of light at small thicknesses, particularly in the 600–1100-nm spectral range [11]. As the NW commonly has a short length of several microns, Si NW array solar cells typically have a low conversion efficiency [7–9].

An efficient way of increasing the efficiency of Si NW solar cell is to enhance its absorption in the 600–1100-nm range via introducing new structures or materials. Recently, a family of methylammonium lead halide perovskites CH_3_NH_3_PbX_3_ (*X* = I, Br, Cl) has attracted great attention for its breakthrough in low-cost high-efficiency solar cells [12–14]. The excellent properties including a direct bandgap, large absorption coefficient, long exciton diffusion length (100~1000 nm) and lifetime, and low-cost processes make perovskite an ideal light absorber [12, 15, 16]. The perovskite materials typically have a high and flat absorption efficiency in a wide spectrum. For example, the most popular perovskite material, CH_3_NH_3_PbI_3_, has a high photo-to-current efficiency of 75–80% across almost the entire spectrum (360–750 nm) [17]. Thus, introducing perovskite into the Si NW array is expected to dramatically enhance the absorption in the visible range and increase the conversion efficiency.

**Fig. 1:**
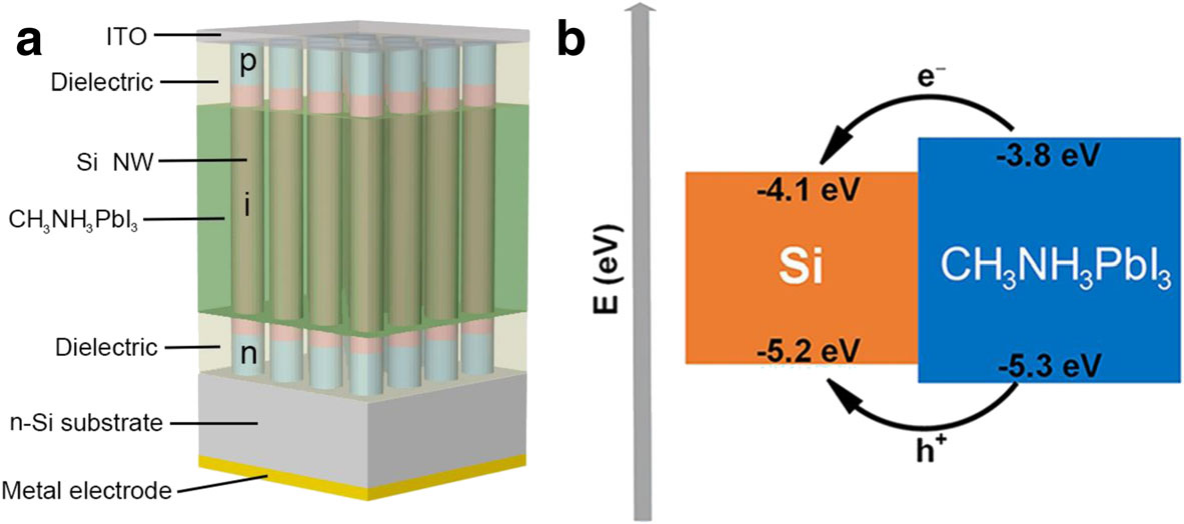
**a** Schematic diagram of the Si NW array/perovskite hybrid solar cell. **b** Band alignment scheme for the Si/CH_3_NH_3_PbI_3_ heterostructure

In this work, we proposed a hybrid solar cell based on Si NW array/perovskite structure, which is schematically illustrated in Fig. 1a. The solar cell consists of a vertical-aligned Si axial p-i-n NW array filled with CH_3_NH_3_PbI_3_ among the intrinsic regions of NWs. CH_3_NH_3_PbI_3_ can be easily inserted into the NW array through a simple spin-coating and immersion process [18]. Both the NWs and CH_3_NH_3_PbI_3_ act as light absorbers and generate electrons and holes. The bandgaps of Si NW and CH_3_NH_3_PbI_3_ are 1.1 and 1.5 eV, respectively. Due to the different absorption coefficient between Si and CH_3_NH_3_PbI_3_ at a certain wavelength, optimal absorption efficiency can be obtained via adjusting the volume ratio between NWs and perovskite. The carriers generated in the NW are directly accelerated in the built-in electric field and transported to the external circuit. According to the band alignment in Fig. 1b, Si/CH_3_NH_3_PbI_3_ forms a type-I heterostructure [19]. The electrons and holes generated in the perovskite naturally fall into the NW channels and then reach the electrodes driven by the built-in electric field. Particularly, due to the long exciton diffusion length and lifetime of perovskite, most of the photo-generated carriers can diffuse to the NW and fall into the channels via optimizing the distance between NWs.

## Methods

The structural similarity without mesoporous structure and the Wannier-type exciton enable to apply an existed device simulator widely used in inorganic solar cells to the perovskite solar cells [20]. Photovoltaic properties of the structure are investigated by using Sentaurus Technology Computer-Aided Design (TCAD). Before simulating the hybrid structure, a Si NW array solar cell without CH_3_NH_3_PbI_3_ is modeled. In the axial p-i-n NW, the intrinsic region is designed to be several times longer than the two doping segments to avoid the recombination losses in the top p segment and enhance the effective absorption. The p and n segments are uniformly doped to 3 × 10^18^ and 1 × 10^17^ cm^− 3^, respectively. The substrate is n-typed Si with a doping concentration of 1 × 10^17^ cm^− 3^. Optical properties of the structure are investigated by using 3D finite difference time domain (FDTD) simulations through the Sentaurus Electromagnetic Wave (EMW) Solver module package. The wavelength-dependent complex refractive index of Si NWs used in the simulations is obtained from Levinshtein’s work [21], while the parameters of the perovskite are obtained from other published work [22, 23]. Incident light is defined with power intensity and wavelength values from a discretized AM 1.5G solar spectrum. The AM 1.5G spectrum is divided into 81 discrete wavelength intervals, from 300 to 1100 nm. The corresponding unpolarized feature of sunlight is modeled by superimposing the transverse electric (TE) and transverse magnetic (TM) mode contributions. The total optical generation under AM 1.5G illumination can be modeled by superimposing the power-weighted single-wavelength optical generation rates. For the electrical modeling, the 3D optical generation profiles are incorporated into the finite-element mesh of the NWs in the electrical tool, which solves the carrier continuity equations coupled with Poisson’s equation self-consistently in 3D. The doping-dependent mobility, bandgap narrowing, and radiative, Auger and Shockley-Reed-Hall (SRH) recombination are taken into consideration in the device electrical simulations. The key parameters for Si and perovskite used in the simulation are shown in Tables 1 and 2, respectively [14, 24]. The performance of the Si NW array solar cell is optimized by tuning the NW diameter, length, and the D/P ratio, which is determined by the period of the square lattice (*P*) and the diameter of NW (*D*).

**Table 1:** Key material parameters for Si

Parameters	Values
Dielectric constant	11.7
Bandgap (eV)	1.1
Electron affinity (eV)	4.05
Minimum mobility (cm^2^/V/s)	52.2 (44.9)
SRH lifetime (ns)	1 (1)
Effective density of states (/cm^3^)	2.54 × 10^19^ (2.54 × 10^19^)
Surface recombination velocity (cm/s)	10^3^ (10^3^)

**Table 2:** Key material parameters for perovskite

Parameters	Values
Dielectric constant	30
Bandgap (eV)	1.5
Electron affinity (eV)	3.93
Electron and hole mobility (cm^2^/V/s)	50, 50
Acceptor concentration (cm^−3^)	2.14 × 10^17^
Donor concentration (cm^−3^)	0
Effective conduction band density (cm^−3^)	2.5 × 10^20^
Effective valence band density (cm^−3^)	2.5 × 10^20^

## Results and Discussion

**Fig. 2:**
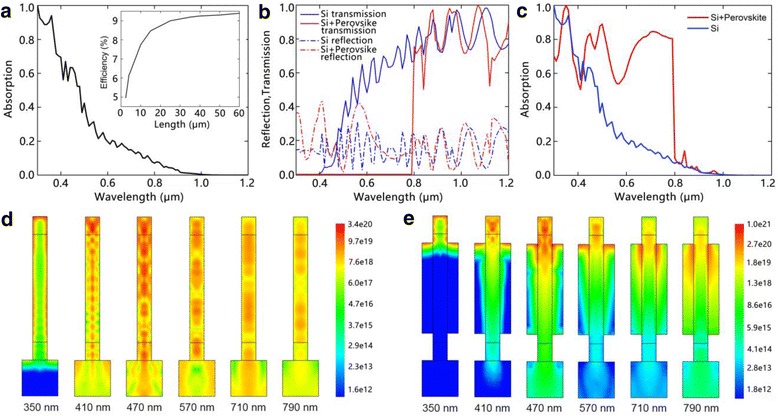
**a** Absorption spectra of the Si NW array. The *inset* shows the dependence of the conversion efficiency of the Si NW array solar cell on the NW length. **b** The transmission and reflection spectra of Si NW array and the hybrid structure. **c** The absorption spectra of Si NW array and the hybrid structure. **d**, **e** Optical generation profiles at different wavelengths for the NW array and hybrid structure, respectively

We first consider a Si NW array solar cell without CH_3_NH_3_PbI_3_. The cell has an optimized NW diameter of 180 nm and D/P ratio of 0.6. Figure 2a shows the absorption spectra of the Si NW solar cell. It can be seen that the solar cell has a high absorption efficiency in the range of 300~500 nm. After 500 nm, the absorption efficiency rapidly drops due to a low absorption coefficient. The inset presents the conversion efficiency of the solar cell with different NW lengths. The efficiency increases with increasing the NW length and saturates at about 9.33% when the NW length exceeds 40 μm. This can be explained by the poor light absorption ability and long carrier diffusion length of Si [25]. Due to the low absorption coefficient of Si in the 600–1100-nm spectral range, the absorption of light is insufficient for short NWs. When the NW length increases, the absorption is enhanced due to an increased absorber volume. In addition, light trapping is also enhanced for longer NWs, which dramatically decreases the light transmission and increases the absorption [2]. As the collection of carriers is efficient due to the long carrier diffusion length of Si, the conversion efficiency is correspondingly increased. When the NW is long enough, the incident light can be sufficiently absorbed, and the conversion efficiency gradually saturates and no longer increases with the NW length. Although the maximum efficiency of Si NW solar cell is comparable to the Si film solar cell, the material cost is much higher due to the considerable long NW length, even considering the lower filling ratio [26].

By introducing CH_3_NH_3_PbI_3_ among NWs, the optical absorption is dramatically improved, as shown in Fig. 2b, c. Benefiting from the large absorption coefficient of CH_3_NH_3_PbI_3_, the hybrid structure has a low transmission and high absorption efficiency in a broad wavelength range of 300~800 nm. Figure 2d, e presents the optical generation profiles of the Si NW array and NW/perovskite hybrid structure at different wavelengths, respectively. For the Si NW array, the photo-generated carriers distribute over the whole structure, even in the substrate. This suggests that the incident photons are not sufficiently absorbed by the NWs with such a length. The absorption peaks at 470 nm and then gradually drops as the wavelength increases, which is in agreement with the low absorption coefficient of Si in the long wavelength range. After introducing CH_3_NH_3_PbI_3_, the optical generation profile substantially changes. An obvious improvement is that the photo-generations are accumulated near the top of the structure, suggesting that the incident photos are sufficiently absorbed by the NWs and perovskite. When the wavelength exceeds 470 nm, an obvious transfer of the photo-generations from Si NWs to the perovskite is observed. This suggests that the strong optical absorption of perovskite sufficiently compensates the poor absorption of Si NWs at longer wavelength, leading to a high absorption efficiency as shown in Fig. 2c.

**Fig. 3:**
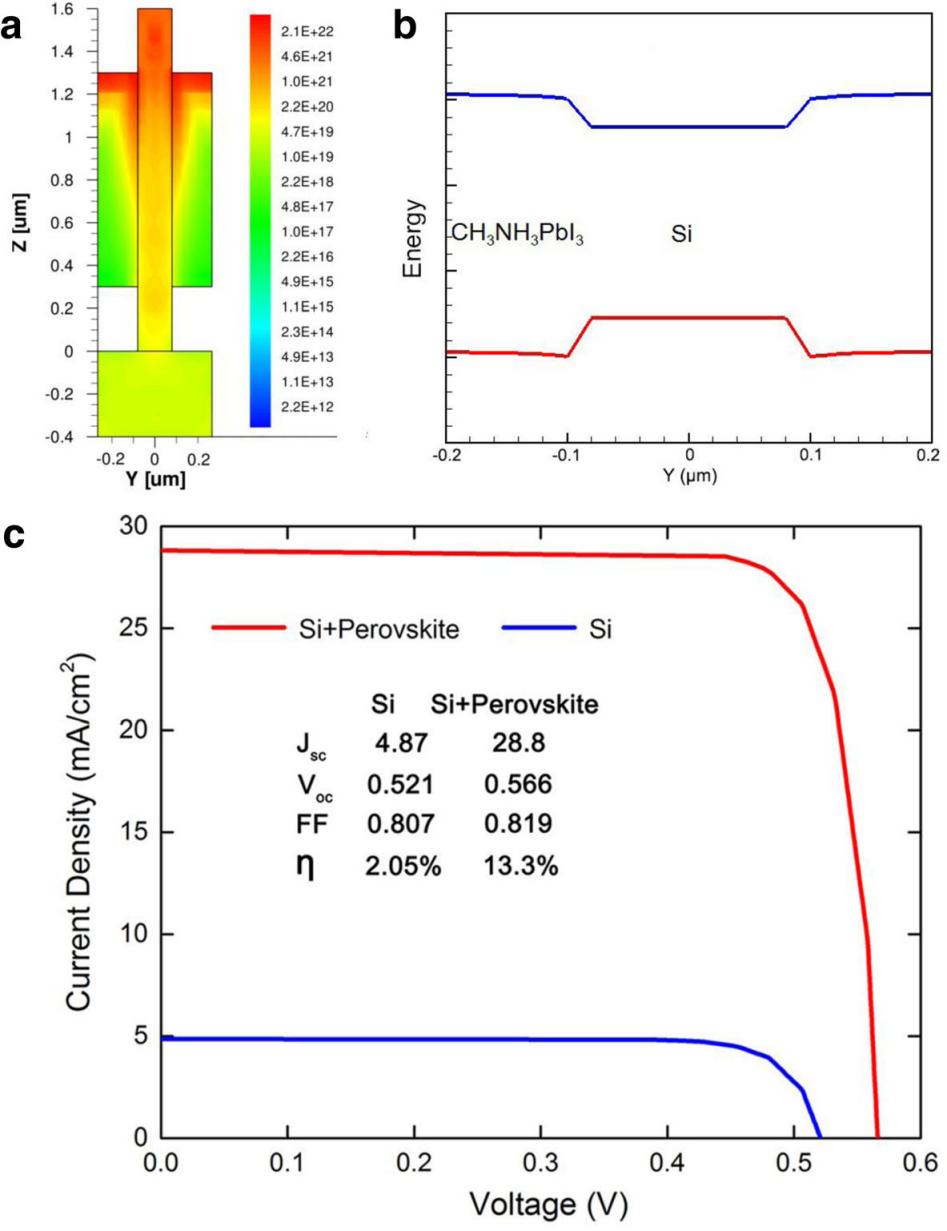
**a** Optical generation profiles of the hybrid structure. **b** Equilibrium band alignment of the hybrid structure. **c** *I*-*V* curves of the hybrid solar cell and Si NW array solar cell under AM 1.5G illumination

Although the perovskite dramatically enhances the absorption of photons, photo-carriers generated in the perovskite cannot directly convert into current due to a lack of built-in electric field and electrodes. Alternatively, the photo-carriers in the perovskite can enter the external circuit via the Si NWs. Figure 3a presents the distribution profile of photo-carriers in the hybrid structure. We can see that most of the photo-carriers accumulate at the top of perovskite as well as the perovskite/Si interface. Due to the concentration difference, photo-carriers diffuse into the Si NWs, followed by being extracted to the electrodes by the built-in electric field in the NW, together with the photo-carriers generated in the NWs. Figure 3b presents the equilibrium band alignment of the cross section of the hybrid structure. We can see that Si and perovskite form a type-I heterostructure, in which Si acts as a well while the perovskite functions as barriers. Thus, both the photo-generated electrons and holes in the perovskite naturally fall into the NW without barriers. Due to the long exciton diffusion length (typically larger than 100 nm) and lifetime of perovskite, most of the photo-carriers in the perovskite can diffuse into the NW and contribute to the current [15, 16]. As Si has a much higher mobility, the recombination of electrons and holes in the NW channel are dramatically reduced in comparison with the perovskite solar cells. Moreover, due to the large surface-to-volume ratio, Si NWs typically contain high density of surface states, which enhance the nonradiative surface recombination and degrade the performance of solar cells [7, 27]. In the hybrid structure, the high-bandgap perovskite may act as a passivation layer, alleviating the surface state effects and enhancing the conversion efficiency of Si NWs [28].

Figure 3c shows the current-voltage (*I*-*V*) of the hybrid solar cell under AM 1.5G illumination. The D/P ratio, NW diameter, and NW length are optimized to be 0.3, 160 nm, and 1.6 μm, respectively. The *I*-*V* curve of the Si NW solar with same parameters is also presented for comparison. The hybrid solar cell yields a large short-circuit current density (*J*_sc_) of 28.8 mA/cm^2^, nearly six times that of the Si NW solar cell. The extra current is attributed to the photo-carriers generated in the perovskite, as well as the reduced nonradiative surface recombination. The open-circuit voltage (*V*_oc_) of the hybrid solar cell is 0.566 V, slightly larger than that of the Si NW solar cell, which is attributed to the improvement of short-circuit current as well as the dark current. As has been reported, *V*_oc_ is proportional to ln(*J*_sc_/*J*_s_) when *J*_sc_/*J*_s_ > > 1, where *J*_s_ is the reverse dark saturation current [29]. For the hybrid structure, *J*_sc_ is dramatically increased due to the enhanced absorption of light, while *J*_s_ is diminished due to the reduction of surface recombination of nanowires passivated by perovskite, resulting in an increase of *V*_oc_. The conversion efficiency (*η*) of the hybrid solar cell is calculated to be 13.3%, nearly seven times that of the Si NW solar cell, and also higher than other organic/Si NW hybrid solar cells [30]. The efficiency is comparable to the best results obtained in direct-bandgap InP and GaAs NW solar cells [2, 31]. As the material and manufacturing cost of Si and perovskite is much lower than III–V compounds, the hybrid solar cell may be more promising in low-cost high-efficiency photovoltaic applications.

**Fig. 4:**
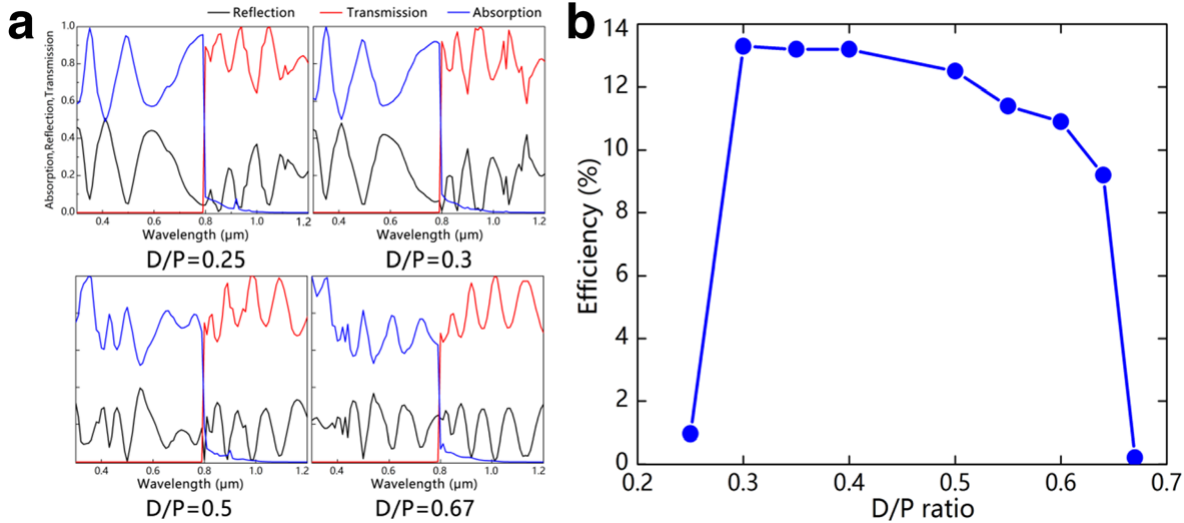
**a** The transmission, reflection, and absorption spectra of the hybrid structure at different D/P ratios. **b** Dependence of the conversion efficiency of the hybrid solar cell on the *D*/*P* ratio

Finally, we investigate the dependence of the conversion efficiency on D/P ratio. It has been widely reported that the D/P ratio has a great influence on the absorption properties of NW array solar cells [32–34]. For the hybrid solar cell, the D/P ratio not only affects the light trapping of the Si NW array but also adjusts the proportion between Si and perovskite for better matching the solar spectra. We have calculated the conversion efficiency with the D/P ratio from 0.25 to 0.67, as shown in Fig. 4. From Fig. 4c, we can see that the device has a high efficiency around 13% in a broad D/P ratio range from 0.3 to 0.5. The low efficiency at high D/P ratio (larger than 0.6) could be mainly attributed to the reduced absorption in the range of 400~800 nm due to the decrease of perovskite proportion, as shown in Fig. 4a. When the D/P ratio decreases to 0.25, the efficiency sharply drops to less than 1%. As the absorption spectra is similar for the D/P ratio of 0.25 and 0.3, the low efficiency at very low D/P ratio should be attributed to the enhanced recombination of electrons and holes generated in the perovskite due to the increase of diffusion distance which severely reduces the total photocurrent.

## Conclusions

In conclusion, we have proposed a Si NW array/perovskite hybrid solar cell and simulated the photovoltaic properties by using Sentaurus TCAD. Benefiting from the large absorption coefficient of CH_3_NH_3_PbI_3_, the hybrid structure has a high absorption efficiency in a broad wavelength range of 300~800 nm. A high *J*_sc_ of 29.1 mA/cm^2^ and remarkable *η* of 13.3% are obtained at a thin absorber thickness of 1.6 μm, which are comparable to the best results of direct-bandgap InP and GaAs NW solar cells. Due to the low material and manufacturing cost of Si and perovskite, the hybrid structure is promising in low-cost high-efficiency solar cells.

## Abbreviation

NW: Nanowire

## References

[1] Duan X, Huang Y, Cui Y, Wang J, Lieber CM (2001). Indium phosphide nanowires as building blocks for nanoscale electronic and optoelectronic devices. Nature.

[2] Garnett E, Yang P (2010). Light trapping in silicon nanowire solar cells. Nano lett.

[3] Krogstrup P, Jørgensen HI, Heiss M, Demichel O, Holm JV, Aagesen M, Nygard J, Morral AF (2013). Single-nanowire solar cells beyond the Shockley–Queisser limit. Nat Photonics.

[4] Wang S, Yan X, Zhang X, Li J, Ren X (2015). Axially connected nanowire core-shell p-n junctions: a composite structure for high-efficiency solar cells. Nanoscale Res Lett.

[5] Kayes BM, Atwater HA, Lewis NS (2005). Comparison of the device physics principles of planar and radial p-n junction nanorod solar cells. J Appl Phys.

[6] Wallentin J, Anttu N, Asoli D, Huffman M, Aberg I, Magnusson MH, Siefer G, Fuss-Kailuweit P, Dimroth F, Witzigmann B, Xu HQ, Samuelson L, Deppert K, Borgström MT (2013). InP nanowire array solar cells achieving 13.8% efficiency by exceeding the ray optics limit. Science.

[7] Tsakalakos L, Balch J, Fronheiser J, Korevaar BA, Sulima O, Rand J (2007). Silicon nanowire solar cells. Appl Phys Lett.

[8] Garnett EC, Yang P (2008). Silicon nanowire radial p-n junction solar cells. J Am Chem Soc.

[9] Gunawan O, Guha S (2009). Characteristics of vapor–liquid–solid grown silicon nanowire solar cells. Sol Energ Mat Sol C.

[10] Adachi MM, Anantram MP, Karim KS (2013). Core-shell silicon nanowire solar cells. Sci Rep.

[11] Atwater HA, Polman A (2010). Plasmonics for improved photovoltaic devices. Nat Mater.

[12] Kojima A, Teshima K, Shirai Y, Miyasaka T (2009). Organometal halide perovskites as visible-light sensitizers for photovoltaic cells. J Am Chem Soc.

[13] Zhou H, Chen Q, Li G, Luo S, Song T, Duan H, Hong Z, You J, Liu Y, Yang Y (2014). Interface engineering of highly efficient perovskite solar cells. Science.

[14] Liu F, Zhu J, Wei J, Li Y, Lv M, Yang S, Zhang B, Yao J, Dai S (2014). Numerical simulation: toward the design of high-efficiency planar perovskite solar cells. Appl Phys Lett.

[15] Sun S, Salim T, Mathews N, Duchamp M, Boothroyd C, Xing G, Sum TC, Lam YM (2014). The origin of high efficiency in low-temperature solution-processable bilayer organometal halide hybrid solar cells. Energy Environ Sci.

[16] Stranks SD, Eperon GE, Grancini G, Menelaou C, Alcocer MJP, Leijtens T, Herz LM, Petrozza A, Snaith HJ (2013). Electron-hole diffusion lengths exceeding 1 micrometer in an organometal trihalide perovskite absorber. Science.

[17] Liu D, Kelly TL (2014). Perovskite solar cells with a planar heterojunction structure prepared using room-temperature solution processing techniques. Nat Photonics.

[18] Burschka J, Pellet N, Moon S, Humphry-Baker R, Gao P, Nazeeruddin MK, Grätzel M (2013). Sequential deposition as a route to high-performance perovskite-sensitized solar cells. Nature.

[19] Wang JT, Ball JM, Barea EM, Abate A, Alexander-Webber JA, Huang J, Saliba M, Mora-Sero I, Bisquert J, Snaith HJ, Nicholas RJ (2014). Low-temperature processed electron collection layers of graphene/TiO2 nanocomposites in thin film perovskite solar cells. Nano Lett.

[20] Minemoto T, Murata M (2014). Device modeling of perovskite solar cells based on structural similarity with thin film inorganic semiconductor solar cells. J Appl Phys.

[21] White TP, Lal NN, Catchpole KR (2014). Tandem solar cells based on high-efficiency c-Si bottom cells: top cell requirements for >30% efficiency. IEEE J Photovolt.

[22] Schneider BW, Lal NN, Baker-Finch S, White TP (2014). Pyramidal surface textures for light trapping and antireflection in perovskite-on-silicon tandem solar cells. Opt Express.

[23] Synopsys (2013). Sentaurus device user guide (version G-2013.03).

[24] Levinshtein M, Rumyantsev S, Shur M (1999). Handbook series on semiconductor parameters, ternary, and quaternary III-V compounds.

[25] Würfel P, Trupke T, Puzzer T, Schäffer E, Warta W, Glunz SW (2007). Diffusion lengths of silicon solar cells from luminescence images. J Appl Phys.

[26] Shah A, Torres P, Tscharner R, Wyrsch N, Keppner H (1999). Photovoltaic technology: the case for thin-film solar cells. Science.

[27] Dan Y, Seo K, Takei K, Meza JH, Javey A, Crozier KB (2011). Dramatic reduction of surface recombination by in situ surface passivation of silicon nanowires. Nano Lett.

[28] Mariani G, Scofield AC, Huang CH, Huffaker DL (2013). GaAs nanopillar-array solar cells employing in situ surface passivation. Nat Commun.

[29] Li N, Lassiter BE, Lunt RR, Wei G, Forrest SR (2009). Open circuit voltage enhancement due to reduced dark current in small molecule photovoltaic cell. Appl Phys Lett.

[30] Wang W, Li X, Wen L, Liu G, Shi T, Duan H, Zhou B, Li N, Zhao Y, Zeng X (2014). Optical and electrical simulations of silicon nanowire array/poly(3hexylthiophene):phenyl-C61-butyric acid methyl ester hybrid solar cell. Appl Phys Lett.

[31] Aberg I, Vescovi G, Asoli D, Vescovi U, Gilboy JP, Sundvall C, Dahlgren A, Svensson KE, Anttu N, Björk MT, Samuelson L (2016). A GaAs nanowire array solar cell with 15.3% efficiency at 1 sun. IEEE J Photovolt.

[32] Li J, Yu H, Wong SM, Li X, Zhang G, Lo PG-Q, Kwong D-L (2009). Si nanopillar array optimization on Si thin films for solar energy harvesting. Appl Phys Lett.

[33] Zhang X, Sun XH, Jiang LD (2013). Absorption enhancement using nanoneedle array for solar cell. Appl Phys Lett.

[34] Wu Y, Yan X, Zhang X, Ren X (2015). Enhanced photovoltaic performance of an inclined nanowire array solar cell. Opt Express.

